# Carnitine o-octanoyltransferase is a p53 target that promotes oxidative metabolism and cell survival following nutrient starvation

**DOI:** 10.1016/j.jbc.2023.104908

**Published:** 2023-06-10

**Authors:** Jack D. Sanford, Derek Franklin, Gabriella A. Grois, Aiwen Jin, Yanping Zhang

**Affiliations:** 1 Department of Radiation Oncology, School of Medicine, University of North Carolina at Chapel Hill, Chapel Hill, North Carolina, USA; 2 Lineberger Comprehensive Cancer Center, School of Medicine, University of North Carolina at Chapel Hill, Chapel Hill, North Carolina, USA; 3 Curriculum in Genetics and Molecular Biology, School of Medicine, University of North Carolina at Chapel Hill, Chapel Hill, North Carolina, USA; 4 Department of Pharmacology, School of Medicine, University of North Carolina at Chapel Hill, Chapel Hill, North Carolina, USA

**Keywords:** p53, CROT, nutrient starvation, oxidative metabolism, cancer

## Abstract

Whereas it is known that p53 broadly regulates cell metabolism, the specific activities that mediate this regulation remain partially understood. Here, we identified carnitine o-octanoyltransferase (CROT) as a p53 transactivation target that is upregulated by cellular stresses in a p53-dependent manner. CROT is a peroxisomal enzyme catalyzing very long-chain fatty acids conversion to medium chain fatty acids that can be absorbed by mitochondria during β-oxidation. p53 induces *CROT* transcription through binding to consensus response elements in the 5′-UTR of *CROT* mRNA. Overexpression of WT but not enzymatically inactive mutant CROT promotes mitochondrial oxidative respiration, while downregulation of CROT inhibits mitochondrial oxidative respiration. Nutrient depletion induces p53-dependent CROT expression that facilitates cell growth and survival; in contrast, cells deficient in CROT have blunted cell growth and reduced survival during nutrient depletion. Together, these data are consistent with a model where p53-regulated CROT expression allows cells to be more efficiently utilizing stored very long-chain fatty acids to survive nutrient depletion stresses.

The tumor suppressor p53 is mutated in roughly 50% of human cancers. The best studied molecular function of p53 is its capacity to regulate numerous target genes through its transcriptional factor activity. Through this activity, p53 is able to regulate many aspects of cellular physiology, including cell cycle progression, apoptosis, ferroptosis, metabolism, redox regulation, and differentiation (1, 2). One critical p53 activity is the regulation of cell death and survival in response to stress. Under mild cell stress, p53 induces cell cycle arrest, upregulates DNA damage response, and promotes metabolic reprogramming in order to facilitate cell survival. Under severe or unresolved cell stress, however, p53 promotes permanent growth arrest through the induction of senescence or eliminates the damaged cells through promoting apoptosis. The exact mechanisms that determine whether p53 promotes a cell survival or cell death following cellular stress, though critically important, remain incompletely understood.

One area of intense research interest is p53-mediated metabolic reprogramming. Loss of p53 function in mice promotes a wide variety of metabolic phenotypes, including altered oxidative and glycolytic metabolism, body fat accumulation, decreased exercise capacity, and impaired insulin signaling, showing p53 as a critical regulator of metabolism (3, 4, 5, 6, 7). p53 is activated in response to several forms of metabolic stress, including nutrient deprivation or nutrient overabundance, through multiple pathways including the ribosomal protein-MDM2-p53 and AMPK-p53 pathways (8, 9, 10). p53 activation during nutrient depletion stress results in metabolic adaptations that have been shown to promote survival of cultured cells as well as in organisms (8, 11). On the cellular level, p53 inhibits glucose uptake and glycolysis while promoting oxidative metabolism of glutamine and fatty acids. Thus the increased survival may result from p53 acting as a metabolic switch that alters cellular metabolism in response to nutrient availability. One such potential prosurvival function of p53 is to promote utilization of stored fatty acids.

p53 has been shown to promote fatty acid oxidation through several distinct mechanisms, including transcriptional upregulation of carnitine palmitoyltransferase 1C (CPT1C), malonyl-CoA decarboxylase (MCD), Lipin 1 (LPIN1), and pantothenate kinase 1 (PANK1) (9, 12, 13, 14). CPT1C promotes mitochondrial fatty acid uptake, facilitating mitochondrial fatty acid oxidation. MCD catalyzes the conversion of malonyl-CoA to acetyl-CoA, which also promotes lipid uptake into the mitochondria (9). LPIN1 has been shown to promote mitochondrial fatty acid oxidation by binding to PCG1α and promoting transcriptional activation of a set of genes that promote mitochondrial fatty acid oxidation (15). PANK1 promotes CoA biosynthesis, which is a critical cofactor for fatty acid metabolism (13). These studies show that p53 promotes mitochondrial fatty acid metabolism through a wide variety of mechanisms.

A recent microarray analysis performed by our laboratory identified carnitine o-octanoyltransferase (CROT) as a putative p53 transactivation target. We report here that cellular stress induces CROT expression in a p53-dependent manner and that CROT plays a critical role for cell survival during nutrient depletion by facilitating the utilization of very long-chain fatty acids (VLCFAs) during mitochondrial oxidative respiration.

## Results

### p53 regulates CROT expression

We conducted a microarray study to identify p53 target genes in a stress-agnostic manner by activating p53 in a mouse embryonic fibroblast cell system. This study identified CROT as a putative p53 target gene. CROT regulates the exchange of CoA for carnitine on medium chain fatty acids (MCFAs) (C8-C14), which can then be further catabolized in the mitochondria through oxidative phosphorylation (OXPHOS). CROT activity is critical for proper oxidation of VLCFAs, because CoA esters must be replaced with carnitine esters prior to mitochondrial import (16). Consistently, previous studies have found that downregulation of CROT results in an accumulation of VLCFAs (17).

**Figure 1:**
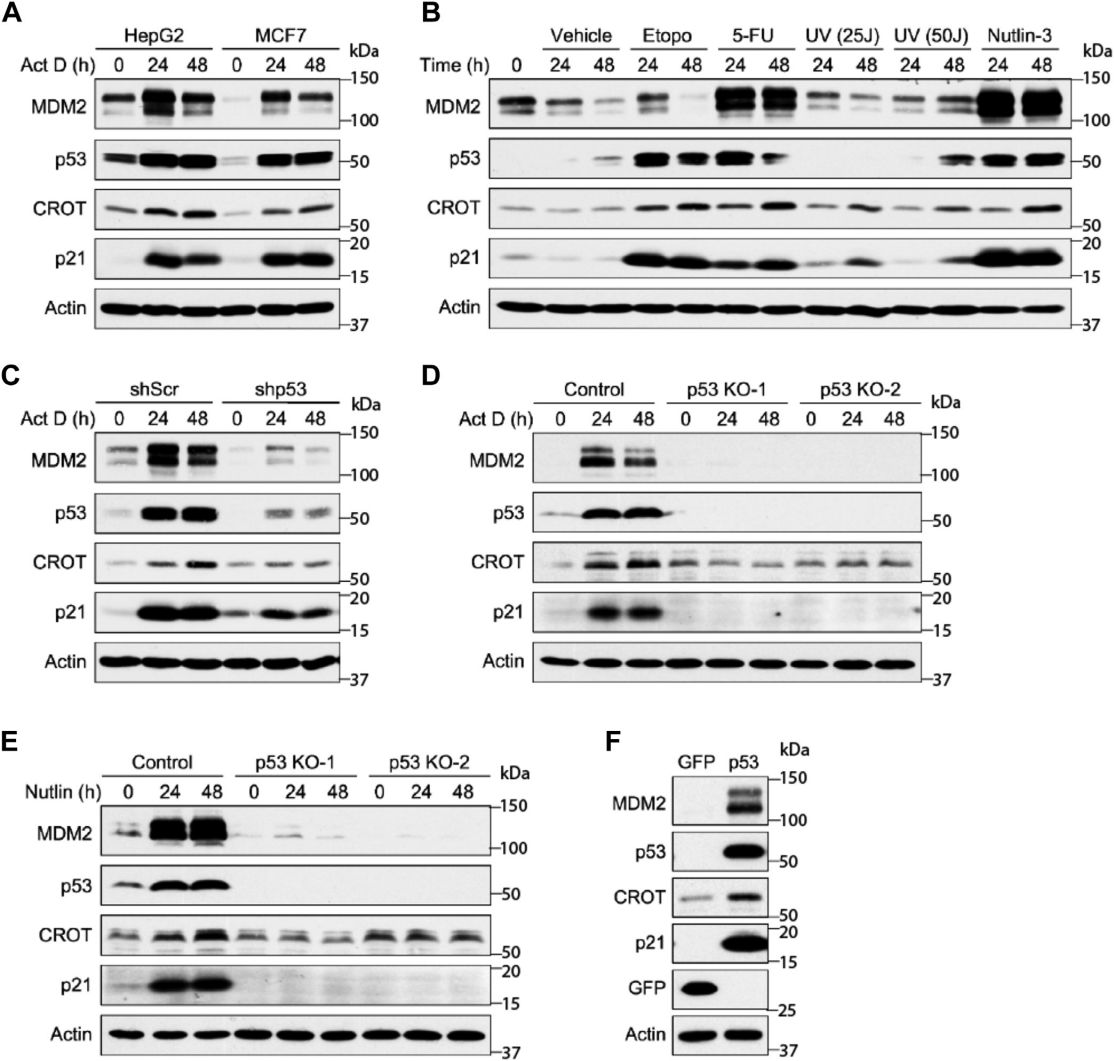
**CROT expression is regulated by p53.***A*, HepG2 and MCF-7 cells were treated with 5 nM ActD for 0, 24, and 48 h prior to harvesting cell lysates for immunoblot analysis. *B*, HepG2 cells were treated with vehicle, etoposide (5 μg/ml), 5-FU (10 μM), UV 25 J/m^2^, UV 50 J/m^2^, or Nutlin-3 (10 μM) for 24 or 48 h prior to cell lysis and immunoblot analysis. *C*, HepG2 cells stably infected with shp53-expressing lentivirus were treated with ActD for 0, 24, and 48 h prior to cell lysis and immunoblotting. *D*, WT p53 HCT116 cells and two CRISPR-mediated p53 KO HCT116 cells were treated with ActD (5 nM) for 0, 24, or 48 h prior to cell lysis and immunoblotting. *E*, WT p53 HCT116 cells and two CRISPR-mediated p53 KO HCT116 cells were treated with Nutlin-3 (10 μM) for 0, 24, or 48 h prior to cell lysis and immunoblotting. *F*, Saos2 cells (p53-null) were infected with adenoviral GFP or p53 for 24 h prior to cell lysis and immunoblot analysis. 5-FU, 5-fluorouracil; CROT, carnitine o-octanoyltransferase.

To verify if p53 regulates CROT expression, p53-positive HepG2 and MCF7 cancer cell lines were treated with low levels (∼5 nM) of actinomycin D (ActD), which activates p53 through induction of ribosomal stress. Following ActD treatment, CROT expression was increased along with p53 targets p21 and MDM2 in both cell lines (Fig. 1*A*). We next investigated whether additional p53-activating treatments similarly promote CROT expression. CROT expression is increased in response to multiple DNA damage conditions inducing etoposide, UV irradiation, and 5-fluorouracil treatment, as well as in response to non-DNA damage treatment by the MDM2 inhibitor nutlin-3 (Fig. 1*B*). These data indicated that CROT expression is responsive to various p53-activating processes.

To determine whether stress-induced CROT expression is p53-dependent, we knocked down p53 in HepG2 cells by shRNA followed by treating the cells with ActD. Downregulation of p53 attenuated ActD-induced CROT expression to a similar degree as the reduction of MDM2 and p21 (Fig. 1*C*). To further demonstrate that p53 is necessary for stress-induced CROT accumulation, we knocked out p53 in HCT116 cells by CRISPR/Cas9 and examined CROT expression after treating the cells with ActD and nutlin-3. In the absence of p53, treating cells with ActD and nutlin-3 did not noticeably induce CROT expression (Fig. 1, *D* and *E*), indicating that stress-induced CROT expression is p53-dependent. Conversely, ectopic overexpression of p53 in p53-null Saos2 cells was sufficient to increase CROT expression (Fig. 1*F*). Taken together, these data showed that CROT protein expression is elevated by p53 in multiple cell types in response to various p53-activating conditions, including ribosomal stress, several types of DNA damage, and pharmacological inhibition of MDM2, as well as by ectopically overexpressed p53.

### CROT is a direct p53 transcriptional target

**Figure 2:**
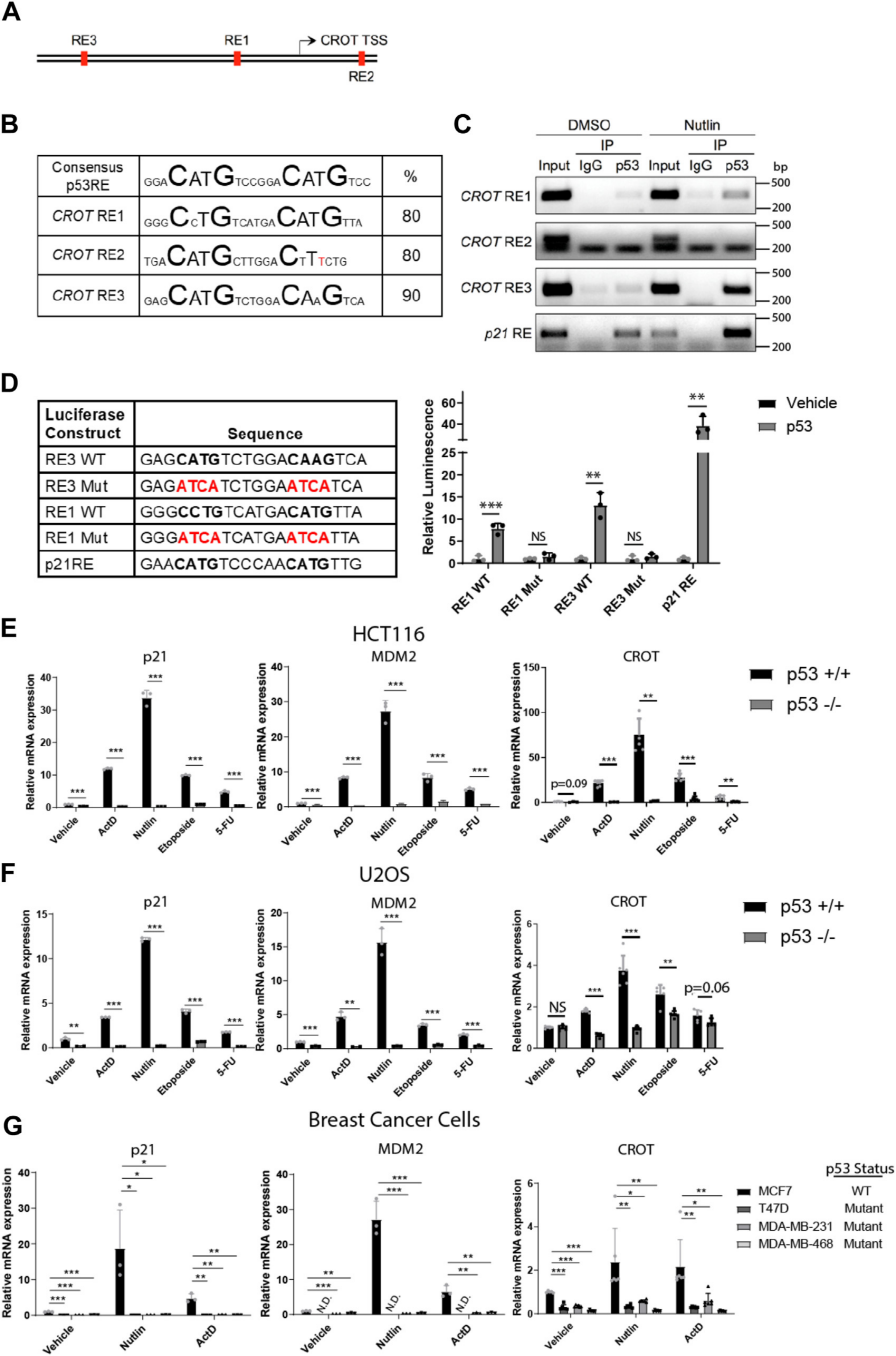
**CROT is a direct p53 transcriptional target.***A*, schematic representation of the portion of chromosome 7 containing CROT with relative locations of p53 response element (RE) and CROT translation start site (TSS) indicated. *B*, comparison of the consensus p53 RE with the three putative REs in the *CROT* promoter region. The essential C and G bases are shown in larger *bold font* with incompatible bases being highlighted in *red*. Percentages represent the base agreement with the consensus sequence with no emphasis for the central C and G bases. *C*, HepG2 cells were treated with nutlin-3 for either 0 or 12 h prior to crosslinking and immunoprecipitation using anti-p53 antibodies. The ChIP-purified DNA was then used as a template for PCR analysis targeting p53REs in either CROT or p21 promoter regions. *D*, luciferase constructs for CROT RE1, RE3, and p21RE were generated by cloning the RE to upstream of the luciferase gene. Mutant constructs for RE1 and RE3 were made by mutating the central CATG bases (shown in *red*). The constructs were then cotransfected with either vehicle or p53 plasmids in Saos2 cells. Relative levels of luminescence (y-axis) are shown. Bars represent mean, points represent individual measurements, and error bars represent SD. (n = 3). *E*, RNA was harvested from p53^+/+^ or p53^−/−^ HCT116 cells treated with either vehicle (1:1000 DMSO), actinomycin D (5 nM), nutlin-3 (10 μM), etoposide (5 μg/ml), or 5-FU (10 μM) for 24 h prior to RT-PCR analysis. Fold increases of mRNA levels compared to actin are shown. Bars represent mean, points represent individual measurements, and error bars represent SD. (n = 3 for p21 and MDM2. N = 6 for CROT). *F*, RNA was harvested from p53^+/+^ or p53^−/−^ U2OS cells treated with either vehicle (1:1000 DMSO), actinomycin D (5 nM), nutlin-3 (10 μM), etoposide (5 μg/ml), or 5-FU (10 μM) for 24 h prior to RT-PCR analysis. Fold increases of mRNA levels compared to actin are shown. Bars represent mean, points represent individual measurements, and error bars represent SD. (n = 3 for p21 and MDM2. N = 6 for CROT). *G*, RNA was harvested from breast cancer cells treated with either vehicle (1:1000 DMSO), nutlin 3 (10 μM), or actinomycin D (5 nM) for 24 h prior to RT-PCR analysis. p53 status of the breast cancer cells is as follows: MCF7 (p53^+/+^), T47D (L194F), MDA-MB-231 (R280K), MDA-MB-468 (R273H). MDM2 mRNA was not detected (N.D.) by qPCR in T47D samples under untreated or treated conditions. Fold increases of mRNA levels compared to actin are shown. Bars represent mean, points represent individual measurements, and error bars represent SD. (n = 3 for p21 and MDM2. N = 6 for CROT). Statistics: Two-tailed unpaired t-tests were used to generate *p*-values. ∗*p* < 0.05; ∗∗*p* < 0.01; ∗∗∗*p* < 0.001. 5-FU, 5-fluorouracil; ChIP, chromatin immunoprecipitation; CROT, carnitine o-octanoyltransferase.

To determine whether p53 directly regulates CROT transcription, we searched the promoter region of *CROT* gene and identified three potential p53 response elements (REs) near the *CROT* transcription start site (Fig. 2*A*). All three putative p53REs showed significant alignment with the p53RE consensus sequence with identity of 80 to 90%, with RE2 has an alternate base at the center of the consensus p53 REs (Fig. 2*B*). Chromatin immunoprecipitation (ChIP) PCR assays demonstrated that p53 binds to *CROT* RE1 and *CROT* RE3 located upstream of the *CROT* transcription start site, while *CROT* RE2 did not specifically interact with p53 (Fig. 2*C*). The functionality of p53 binding to *CROT* RE1 and *CROT* RE3 was then evaluated by luciferase reporter assays. The WT RE1 and RE3 luciferase constructs exhibited significant luminescence in the presence of exogenous p53, indicative of RE1 and RE3 response to p53 expression (Fig. 2*D*). Importantly, the response was ablated by mutations of the central portion in each of the REs (RE1 Mut and RE3 Mut) (Fig. 2*D*). Furthermore, reverse transcription-quantitative polymerase chain reaction (RT-qPCR) analysis of HCT116 (Fig. 2*E*) and U2OS (Fig. 2*F*) cancer cell lines demonstrated p53-dependent CROT mRNA is increased along with the p53 target genes *p21* and *MDM2* in response to several p53-activating conditions. In order to determine whether CROT is regulated by cancer-associated mutant p53, a panel of breast cancer cells with varying p53 status was analyzed. p53 was activated by nutlin-3 or ActD in MCF7 (p53^+/+^), T47D (L194F), MDA-MB-231 (R280K), and MDA-MB-468 (R273H) breast cancer cells. Following p53 activation, p21, MDM2, and CROT mRNA levels were significantly increased in p53^+/+^ MCF7 cells (Fig. 2*G*). Many studies have shown that cancer-associated p53 mutation severely blunts the transcriptional activities of p53. Consistently, induction of these target genes was severely abrogated in T47D, MDA-MB-231, and MDA-MB-468 cells, indicating that p53 mutation significantly inhibits induction of CROT under these conditions. Overall, these data indicated that p53 promotes CROT expression by direct transcriptional regulation mediated by binding two p53REs located near the *CROT* promoter region.

### CROT promotes oxidative metabolism

**Figure 3:**
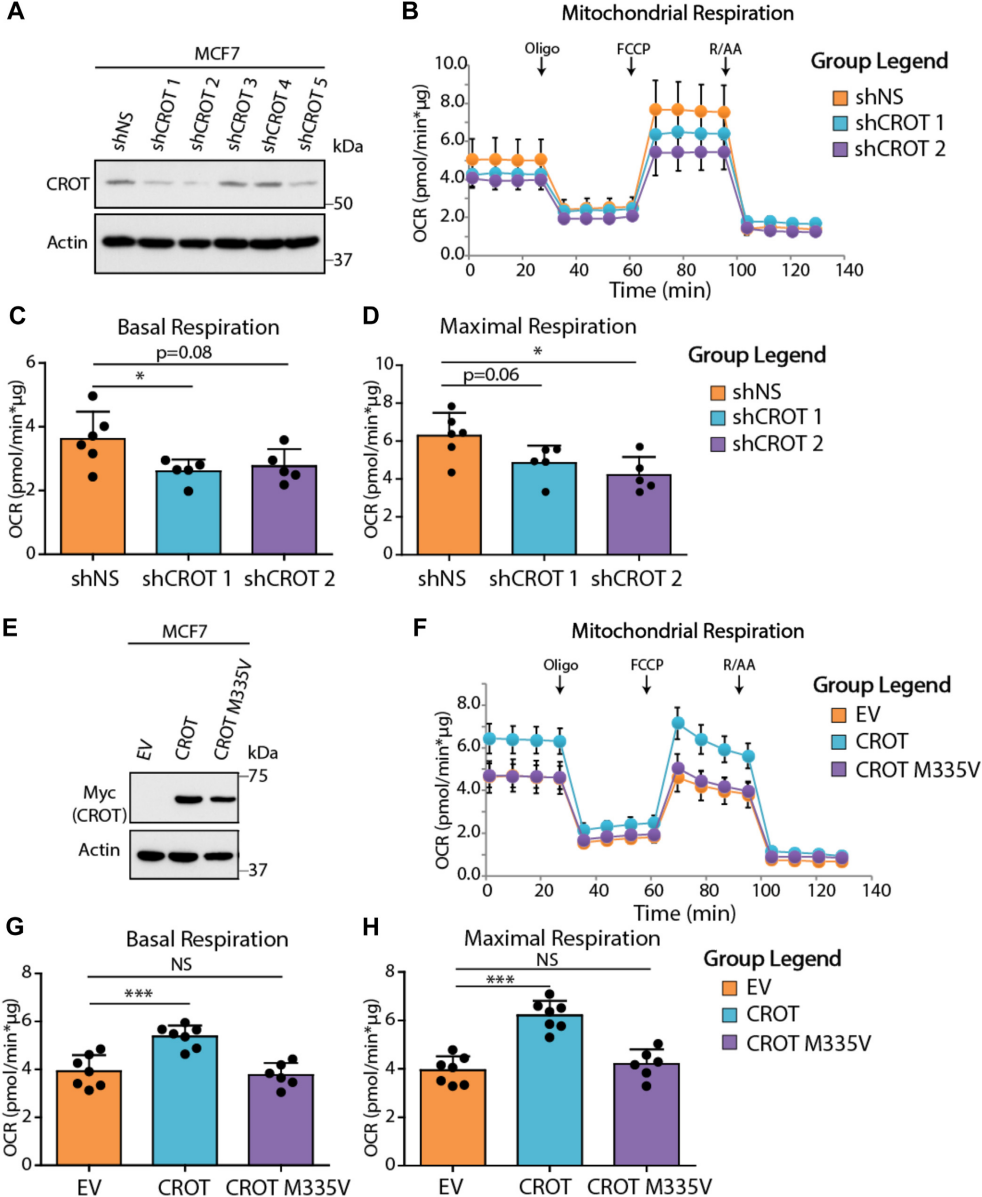
**CROT promotes mitochondrial oxidative metabolism.***A*, MCF7 cells stably transduced with lentiviral shRNA targeting GFP (shNS) or CROT (shCROT) were lysed and protein levels were determined using Western blot analysis. *B*, oxygen consumption rate (OCR) of MCF7 stable cells was determined using the Seahorse Bioanalyzer XFe24 instrument. 40,000 cells were plated in each well 24 h before analysis. At the indicated time points, oligomycin (1.5 μM final concentration), FCCP (0.5–2.5 μM final concentration), and Rotenone + antimycin A (0.5 μM final concentration) were added to each well during live cell analysis. After Seahorse analysis, cells were lysed in 0.5% NP-40 and protein concentration was determined. Data were then normalized to total protein quantity in each well. Points represent mean values, while error bars represent standard deviation. n = 5 to 6 wells were analyzed. *C* and *D*, the data in (*B*) were quantified for basal (*C*) and maximal (*D*) OCR rates. Bars represent mean, points represent individual measurements, and error bars represent SD. n = 5 wells were analyzed. *E*, MCF7 stable cells stably transfected with pcDNA3 vectors expressing no insert (empty vector, EV), myc-CROT, or myc-CROT^M335V^ were lysed, and protein levels were determined using Western blot analysis. *F*, OCR of MCF7 stable cells was determined using the Seahorse Bioanalyzer XFe24 instrument. 40,000 cells were plated in each well 24 h before analysis. At the indicated time points, oligomycin (1.5 μM final concentration), FCCP (0.5–2.5 μM final concentration), and Rotenone + antimycin A (0.5 μM final concentration) were added to each well during live cell analysis. After Seahorse analysis, cells were lysed in 0.5% NP-40 and protein concentration was determined. Data were then normalized to total protein quantity in each well. Points represent mean values, while error bars represent SD. n = 5 to 6 wells were analyzed. *G* and *H*, the data in (*F*) were quantified for basal (*G*) and maximal (*H*) OCR rates. Bars represent mean, points represent individual measurements, and error bars represent SD. n = 5 wells were analyzed. Statistics: Two-tailed unpaired t-tests were used to generate *p*-values. ∗*p* < 0.05; ∗∗∗*p* < 0.001. CROT, carnitine o-octanoyltransferase.

Given that p53 broadly promotes oxidative metabolism and that CROT catalyzes the rate-limiting step in VLCFA oxidation, we hypothesized that CROT promotes oxidative metabolism by enhancing VLCFA oxidation. To assess the role of CROT in oxidative metabolism, we generated a set of CROT shRNA-expressing MCF7 stable cell lines (Fig. 3*A*). We analyzed the oxygen consumption rate (OCR), a measurement for oxidative metabolism, for these MCF7 stable cells in real time using the Seahorse Bioanalyzer. Consistent with our hypothesis, Seahorse analysis revealed that CROT knockdown resulted in the suppression of OCR under both basal and maximal respiratory conditions (Fig. 3, *B*–*D*), indicating that CROT is necessary for cells to undergo proper mitochondrial oxidative respiration. We also tested whether increased levels of CROT might promote mitochondrial respiration by generating cells that stably overexpress WT CROT and CROT^M335V^ mutant that has reduced enzymatic activity due to an M335>V335 mutation in the substrate-binding pocket of CROT (18) (Fig. 3*E*). Cells overexpressing WT CROT demonstrated increased basal and maximal OCR, and this increase in oxidative capacity was dependent on CROT enzymatic activity as the CROT^M335V^ mutant showed much reduced capability to change the rate of mitochondrial respiration (Fig. 3, *F*–*H*). Hence, CROT plays an important role in mitochondrial respiration, and increase in CROT level promotes mitochondrial respiration.

### CROT facilitates cell growth and survival during nutrient starvation

**Figure 4:**
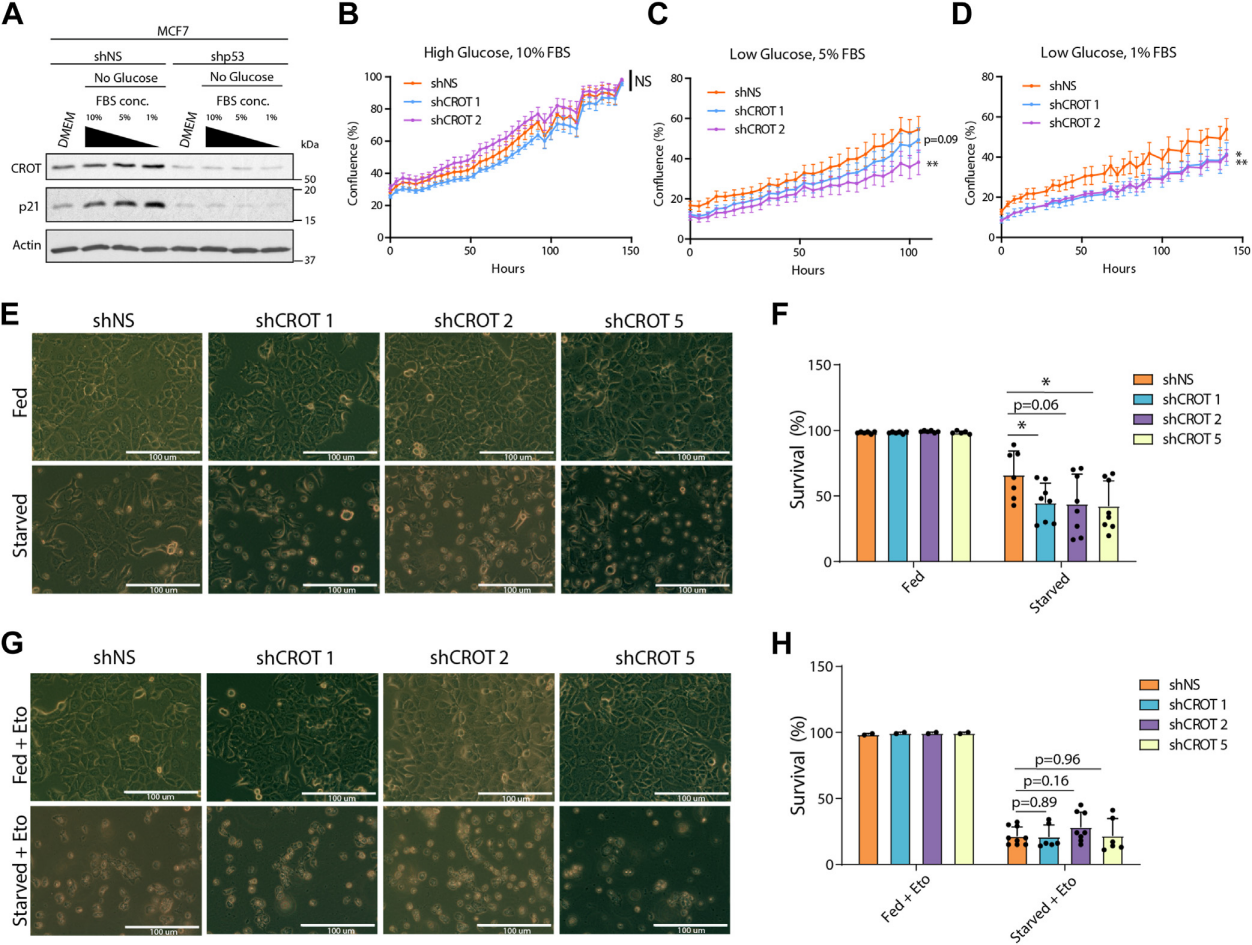
**CROT promotes cell growth and survival following nutrient starvation stress.***A*, MCF7 cells stably transfected with shRNA targeting nonspecific sequences (NS) or p53 were treated with the indicated starvation conditions for 24 h prior to cell lysis and Western blot analysis. *B*–*D*, MCF7 cells stably transfected with shRNA targeting nonspecific sequences (NS) or CROT were plated 40,000 per well in the indicated media containing *high*, *medium*, and *low* concentrations of glucose. Cell growth was analyzed using the Incucyte S3 Live-Cell Analysis System (Sartorius). Four images of live cells were taken every 6 h, and cell confluence was determined using optical density using the Incucyte software package. Points represent mean, while error bars represent SD. Areas under the curve calculations were then used to determine statistical significance. Statistical significance was determined using area under the curve calculations for each well. n = 4 wells were analyzed for each condition. *E* and *F*, MCF7 cells stably transfected with shRNA targeting nonspecific sequences (NS) or CROT were incubated in DMEM (fed), starvation media (0% glucose, 0% pyruvate, 0% FBS). Representative images of cells were taken 48 h after treatment (*E*), or cell survival was determined 48 h after treatment using trypan blue exclusion (*F*). Bars represent mean, points represent individual measurements, and error bars represent SD. n = 6 wells were analyzed under fed conditions. n = 7 to 8 wells were analyzed under starvation conditions. *G* and *H*, MCF7 cells stably transfected with shRNA targeting nonspecific sequences (NS) or CROT were incubated in DMEM supplemented with 100 μM etomoxir (Fed + Eto) or starvation media (0% glucose, 0% pyruvate, 0% FBS) supplemented with 100 μM etomoxir (starved + Eto). Representative images of cells were taken 48 h after treatment (*F*) or cell survival was determined 48 h after treatment using trypan blue exclusion (*H*). Bars represent mean, points represent individual measurements, and error bars represent SD. n = 2 wells were analyzed under fed conditions. n = 6 to 9 wells were analyzed under starvation conditions. Statistics: Two-tailed unpaired t-tests were used to generate *p*-values. ∗*p* < 0.05; ∗∗*p* < 0.01. CROT, carnitine o-octanoyltransferase; DMEM, Dulbecco’s modified Eagle’s medium.

p53 activation during nutrient depletion stress results in metabolic adaptations that have been shown to promote survival of cells and animals (8, 11). We hypothesized that p53-mediated CROT transactivation may contribute to metabolic adaptation and cell survival by allowing cells to utilize stored VLCFA in response to nutrient deprivation. We first investigated whether p53 promotes CROT expression in response to nutrient deprivation. Consistently, nutrient starvation resulted in p53-dependent CROT expression in parental MCF7 cells but not in MCF7 cells without p53 (Fig. 4*A*). This induction of CROT occurred in a dose-dependent manner, in which the level of CROT expression correlated with the severity of the starvation conditions. We next examined whether CROT is important to promote cell growth under nutrient deprivation conditions. CROT knockdown did not noticeably affect the growth rate of cells under high glucose and 10% FBS conditions (Fig. 4*B*). However, under low glucose and reduced FBS concentrations, CROT knockdown blunted cell growth (Fig. 4, *C* and *D*), indicating that CROT provides a growth advantage for cells under nutrient deprivation conditions but does not do so under nutrient abundant conditions.

We next investigated whether CROT is important for cell survival under extreme starvation conditions. We treated parental MCF7 cells and MCF7 cells with stable CROT knockdown under both fed (Dulbecco’s modified Eagle’s medium, 10% FBS) and complete starvation (0% glucose, 0% pyruvate, 0% FBS) conditions and determined cell survival by microscopy and trypan blue exclusion. Complete nutrient depletion resulted in significant cell death in CROT knockdown MCF7 cells compared to moderate cell death in parental MCF7 cells (Fig. 4*E*), indicating that CROT plays an important role for cell survival following nutrient depletion conditions. Quantification of cell survival by trypan blue exclusion revealed that CROT knockdown resulted in significantly decreased survival compared to nontargeting shRNA treatment (Fig. 4*F*). Consistently, U2OS cells stably overexpressing CROT had increased survival under glucose deprivation conditions when compared to control cells (Fig. S1).

To determine whether CROT-promoted cell survival was due to increased mitochondrial fatty acid OXPHOS, we treated cells with the fatty acid oxidation inhibitor etomoxir to block fatty acid OXPHOS (19). While etomoxir treatment of cells growing under fed conditions resulted in no visible cell death regardless of their CROT status, etomoxir treatment of cells growing under starvation conditions resulted in substantial cell death and, importantly, addition of etomoxir ablated the observed differences in survival between parental MCF7 cells and CROT knockdown MCF7 cells (Fig. 4, *G* and *H*), indicating that CROT-promoted cell survival is dependent on increased fatty acid OXPHOS in this context. Together, these studies show that nutrient deprivation results in p53-dependent activation of CROT expression and that CROT expression promotes cell growth and survival under nutrient deprivation and starvation conditions by enhancing fatty acid OXPHOS.

## Discussion

**Figure 5:**
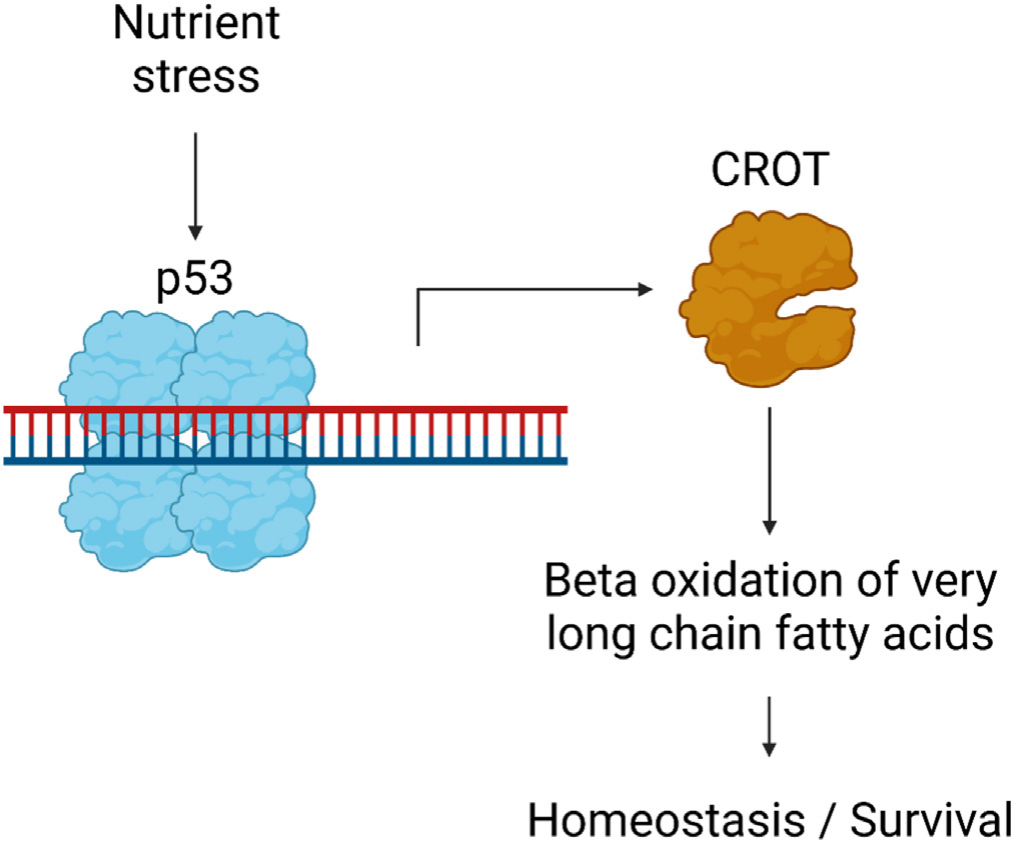
**A hypothetical model depicting the p53-CROT signaling pathway.** Following nutrient depletion stress, p53 transactivates *CROT* gene expression. CROT promotes mitochondrial oxidative metabolism, likely through increasing the rate of very long chain fatty acid (VLCFA) oxidation. Increased utilization of stored long chain fatty acids helps the cell survive during conditions of nutrient stress. CROT, carnitine o-octanoyltransferase.

Our study reveals that CROT is a p53 transactivation target that promotes mitochondrial oxidative metabolism and cell survival following nutrient depletion stress. CROT has known enzymatic activity that promotes VLCFA oxidation by allowing for peroxisomal export and mitochondrial import of VLCFA degradation products (16, 17, 18). To our knowledge, *CROT* is the first p53 target gene that is involved in peroxisomal VLCFA oxidation. Overall, our data support a hypothetical model in which cell stress results in p53-dependent CROT induction that increases utilization of stored VLCFAs. VLCFA oxidation generates MCFAs that can then be more easily taken up by the mitochondria for energy generation or biosynthetic purposes, allowing the cell to respond to nutrient starvation stresses (Fig. 5). This model suggests CROT transactivation as a homeostatic prosurvival activity of p53 under nutrient deprivation conditions. Our findings expand on studies that have shown that p53 plays an important role in fatty acid metabolism. Through the transactivation of CPT1, MCD, LPIN1, and PANK1, p53 is able to promote mitochondrial uptake and oxidation of fatty acid substrates (9, 12, 13, 14). We hypothesize that upregulation of CROT allows p53 to utilize VLCFA in addition to cytosolic MCFAs that can easily be taken up by the mitochondria. The source of these VLCFA moieties remains unclear as VLCFAs are synthesized de novo and can be imported from cell culture media (20, 21, 22, 23). Upregulation of VLCFA oxidation is also consistent with the general model of p53-mediated regulation of the cellular metabolism, in which p53 promotes mitochondrial oxidative metabolism of glutamine and lipids while inhibiting glucose uptake and glycolysis (1, 5, 24, 25).

Our study shows that CROT knockdown results in decreased cellular survival under nutrient deprivation conditions. Given that tumors are frequently under severe metabolic stress, it is possible that CROT could be a therapeutic target for the treatment of cancer. Previous studies of the role of CROT in cancer have postulated diverse opinions regarding whether CROT promotes or inhibits cancer progression. One recent study has revealed that CROT is upregulated in a mouse model of melanoma circulating tumor cells (26). Consistent with our results, this study revealed that CROT knockdown resulted in decreased survival of cells grown in suspension and reduced metastatic propensity of melanoma cells. Another study has shown that CROT expression was downregulated in ovarian cancer and that CROT has tumor-suppressive activities in ovarian cancer cell lines (27). The Cancer Genome Atlas analysis has revealed that TP53 mutations are observed in 96% of ovarian cancer cases (28). Our data show that CROT is a p53 transcriptional target, and therefore the reduced CROT expression in ovarian cancer may be due to loss of p53-mediated CROT transactivation. However, it is still possible that CROT has context-dependent tumor-suppressive activities. Future studies will be required to determine the contexts in which CROT acts as a tumor suppressor or therapeutic target.

## Experimental procedures

### Cell culture and reagents

HepG2, MCF-7, U2OS, and HCT116 cells were maintained in Dulbecco's Modified Eagle Medium supplemented with 10% fetal bovine serum, 100 μg/ml penicillin, and 100 μg/ml streptomycin in the presence of 5% CO2 in a humidified incubator. Cells were treated with nutlin-3 (Selleckchem), ActD (Sigma), etoposide (Sigma), 5-fluorouracil (the University of North Carolina pharmacy). Mammalian protein extraction was accomplished using either NP-40 or SDS lysis buffer.

### ChIP assay

HepG2 cells expressing endogenous p53 were subjected to ChIP assays according to the instructions recommended by the manufacturer (Quick ChIP kit, Novus Biological). Briefly, cells were treated with either 0 or 10 μM nutlin-3 12 h before crosslinking with 1% formalin. After cell lysis, the lysates were sonicated (Branson) to generate ∼1000-bp fragments. Goat anti-human p53 FL-393 antibody and protein-A beads were used to immunoprecipitate p53–DNA complexes. Immunoprecipitated DNA was utilized as a template for PCR reactions consisting of 40 cycles of 95 °C for 30 s, 60 °C for 30 s, and 72 °C for 1 min and further analyzed with QuantStudio 6 Flex Real-Timer PCR System (Applied Biosystems) using the following primers:

P21REF 5′ – CCACTGAGCCTTCCTCACAT - 3′

P21RER 5′- TCTGACTCCCAGCACACACT - 3′

CROT RE 1 F 5′ – AGCCTCACTTCCCTTCAGGT - 3′

CROT RE 1 R 5′ – TATGCCGCAGCACACTACAT – 3′

CROT RE 3 F 5′ – GATAGCTGGGCATTTCATCTGCATAAAGC – 3′

CROT RE 3 R 5′ – GGGTCTCCACCCTTGAGGAGG – 3′

### Luciferase plasmids

The pGL3 basic vector was utilized to subclone the identified p53RE’s from CROT upstream of the firefly luciferase gene in each vector using the following insert oligos. The 5′ side of each forward primer and the 3′ side of each reverse primer had a Kpn1 RE site. The 5′ side of each reverse primer and the 3′ side of each forward primer had a Hind III RE site. For the mutant constructs, the essential CATG bases in the putative p53RE were mutated according to the oligo sequences shown below.

CROTRE3wtF 5′-CAGGGTACCGGGCAGAACTTTGCTGAGCATGTCTGGACAAGTCACCTGTGAAGCCAAGCTTGAC-3′

CROTRE3wtR 5′-GTCAAGCTTGGCTTCACAGGTGACTTGTCCAGACATGCTCAGCAAAGTTCTGCCCGGTACCCTG-3′

CROTRE3mutF 5′-CAGGGTACCGGGCAGAACTTTGCTGAGATCATCTGGAATCATCACCTGTGAAGCCAAGCTTGAC-3′

CROTRE3mutR 5′-GTCAAGCTTGGCTTCACAGGTGATGATTCCAGATGATCTCAGCAAAGTTCTGCCCGGTACCCTG-3′

CROTRE1wtF 5′-CAGGGTACCGACAGACAAAGGAATGGGGCCTGTCATGACATGTTAATTTGAAATCAAGCTTGAC-3′

CROTRE1wtR 5′-GTCAAGCTTGATTTCAAATTAACATGTCATGACAGGCCCCATTCCTTTGTCTGTCGGTACCCTG-3′

CROTRE1mutF 5′- CAGGGTACCGACAGACAAAGGAATGGGGATCATCATGAATCATTAATTTGAAATCAAGCTTGAC-3′

CROTRE1mutR 5′- GTCAAGCTTGATTTCAAATTAATGATTCATGATGATCCCCATTCCTTTGTCTGTCGGTACCCTG-3′

p21REF 5′-CAGGGTACCGCTTTCTGGCCGTCAGGAACATGTCCCAACATGTTGAGCTCTGGCAAGCTTGAC-3′

p21RER 5′-GTCAAGCTTGCCAGAGCTCAACATGTTGGGACATGTTCCTGACGGCCAGAAAGCGGTACCCTG -3

### Antibodies

The following antibodies were purchased commercially: rabbit anti-mouse anti-MDM2 2A10 (University of North Carolina Tissue Culture and Molecular Biology Support Facility), mouse anti-actin (Neomarkers, ProteinTech), rabbit polyclonal (H300) anti-CROT (Santa Cruz), rabbit polyclonal anti-CROT (ProteinTech), anti-p53 DO.1 (Neomarkers) and goat anti-p53 FL393 (Santa Cruz), mouse anti-myc (9E10 hybridoma clone). Rabbit anti-p21 (C-19) was generously provided by Dr Yue Xiong (UNC).

### Lentivirus-based shRNA treatment

Lentivirus-based shRNA constructs were purchased from Open Biosystems for human p53 (TRCN0000003753, TRCN0000003754, TRCN0000003755, TRCN0000003756, and TRCN0000003757). shRNA constructs were cloned into the lentivirus-based pLKO.1 vector and were cotransfected into HEK293T cells along with the appropriate packaging vectors to produce infective virions.

### RT-qPCR

Total RNA was prepared from cell lines using RNeasy mini kit (Qiagen). RNA concentration was determined with a NanoDrop spectrophotometer (Thermo Fisher Scientific, NanoDrop 2000c). Complementary DNA was synthesized using Superscript III reverse transcriptase (18080-051, Invitrogen). qRT-PCR was performed with SYBR Green probes using the Applied Biosystems 7900HT Fast Real-Time PCR system. Results were expressed as the fold-change in transcript levels relative to actin.

Actin RTPCR: F, AGAAAATCTGGCACCACACC; R, CTCCTTAATGTCACGCACGA

MDM2 RTPCR: F, GGTGGGAGTGATCAAAAGGA; R, CCTGATCCAACCAATCACCT

CROT RTPCR: F, GCTGGGGTGACAAATCCTAT; R, CAGGGTGTCCATGAAGTCTG

p21 RTPCR: F, GTCAGAACCCATGCGGCAGCAAG; R, CAGGTCCACATGGTCTTCCTCTG

### CRISPR/Cas9 deletion

CRISPR/Cas9 constructs were designed and assembled based on previous reports. Briefly, a high-scoring guide RNAs (gRNAs) was identified using the CRISPR design tool developed by the Feng Zhang lab. CROT exon 1 and p53 exons 3 and 5 were used as input to identify gRNAs, and the gRNAs were synthesized, annealed, and cloned into the PX260 plasmid. gRNA-annealed oligonucleotides included the following:

p53 exon3: Fwd, CACCGTCCTCAGCATCTTATCCGAG; Rev, AAACCTCGGATAAGATGCTGAGGAC

p53 exon5: Fwd, CACCGCCATTGTTCAATATCGTCCG; Rev, AAACCGGACGATATTGAACAATGGC

The CRISPR constructs were transiently transfected into cells for 24 h, after which puromycin was added for 48 to 72 h to select CRISPR transfectants. After selection, individual clones were isolated. After 2 weeks, colonies were screened for the absence of p53, and KO clones were used for subsequent p53 analyses.

### Seahorse bioanalyzer

OCR was analyzed in MCF7 cells stably transfected with the indicated constructs using the Seahorse Bioanalyzer XFe24 instrument. Forty thousand MCF7 cells were plated in each well the day before analysis. One hour before analysis, the wells were washed with 1 ml Seahorse media. Seahorse XF base media was supplemented with glucose (10 mM), pyruvate (1 mM), and glutamine (2 mM). The media was then aspirated, and 500 μl fresh Seahorse media was added. Cells were then incubated at 37 °C at ambient CO_2_ for 1 h before being analyzed. During Seahorse analysis, at the indicated time points, oligomycin (1.5 μM final concentration), FCCP (0.5–2.5 μM final concentration), and Rotenone + antimycin A (0.5 μM final concentration) were added to each well during live cell analysis. After Bioanalyzer analysis, the Seahorse media was carefully aspirated from each well. The plate was then centrifuged for 1 min at 500 rpm in order to pool any residual media in the well. Media was then carefully aspirated once more before the cells were lysed in 10 to 20 μl of 0.5% NP-40–based cell lysis buffer. Protein concentration was then measured for each well and used to determine total protein levels for each well. Total protein amount for each well was then used to normalize the Seahorse data using the ‘normalize’ function in the Wave software (Agilent) (https://www.agilent.com/en/products/cell-analysis/cell-analysis-software/instrument-software/wave-controller-2-6). Normalized data were then exported to Microsoft excel using the Seahorse XF cell mito stress test report generator. The data in the ‘assay parameters per well’ tab was then used for downstream statistical analysis.

### Cell growth rate

Forty thousand MCF7 cells were plated in each well of a 24-well plate. The following day, the media was changed to the denoted glucose and FBS concentration. Cell growth was then measured using the Incucyte S3 Live-Cell Analysis System (Sartorius). Four images of live cells were taken every 6 h, and cell confluence was determined using optical density using the Incucyte software package.

### Cell survival

Two lakh fifty thousand MCF7 cells were plated into each well of a 6-well plate. The following day, media was changed to fresh media (fed), starvation media (0% glucose, 0% pyruvate, 0% FBS), or starvation media with etomoxir (100 μM). After 48 h of starvation, cells were analyzed using white light microscopy or using trypan blue exclusion.

## Data availability

All of the data are contained within the manuscript and the supporting information. All primary data are available upon request.

## Supporting information

This article contains supporting information.

## Conflicts of interest

The authors declare that they no conflicts of interest with the contents of this article.
