# Supplementary material for: Carnitine o-octanoyltransferase is a p53 target that promotes oxidative metabolism and cell survival following nutrient starvation
Source: J Biol Chem. 2023 Jun 10;299(7):104908. doi: 10.1016/j.jbc.2023.104908 (PMC10339192; doi:10.1016/j.jbc.2023.104908)
Supplement: Supporting Figure S1 [file mmc1.pdf]

Figure S1

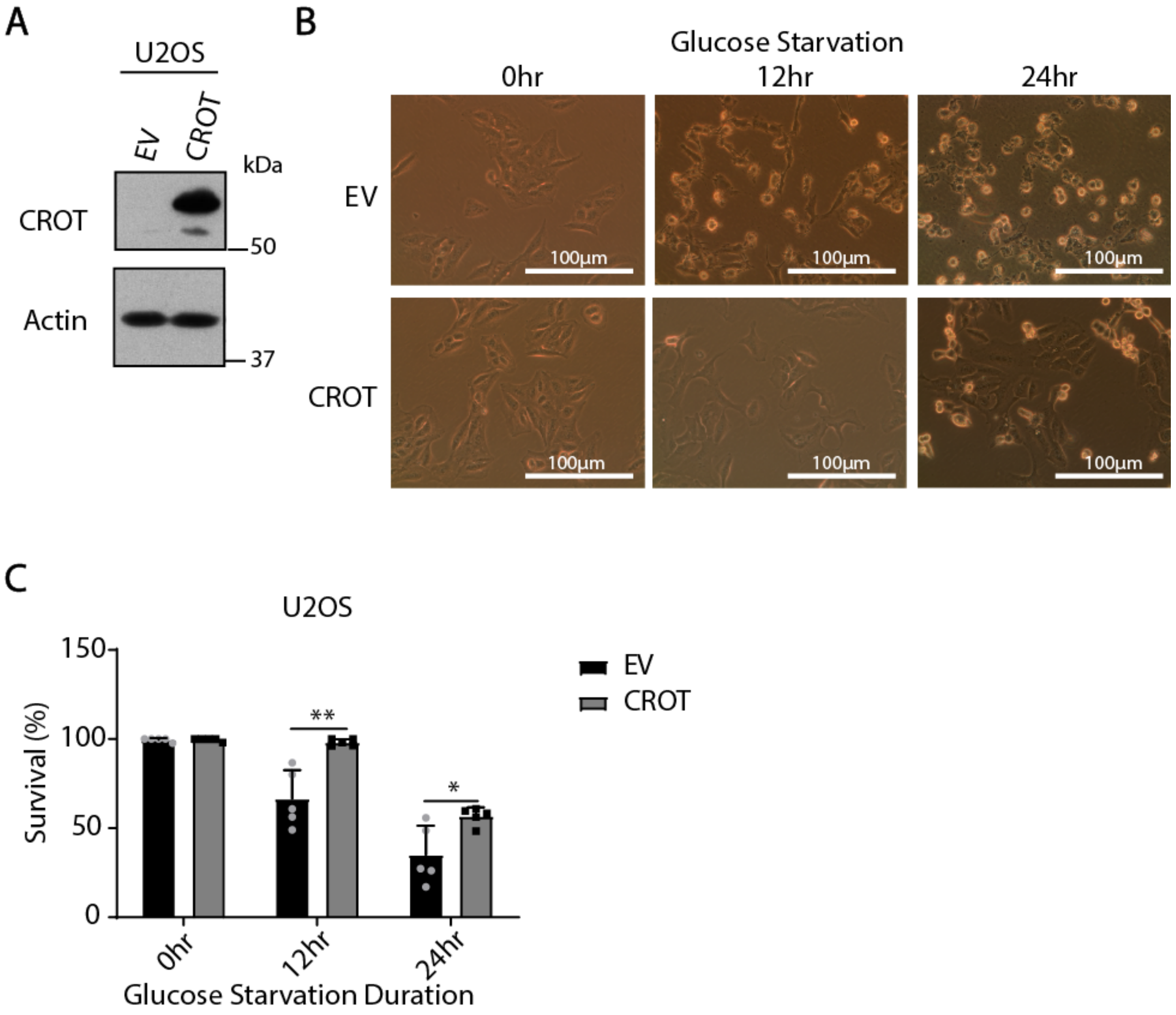

Figure S1: CROT-mediated cell survival in U2OS cells

A) U2OS cells stably expressing empty vector (EV) or Myc-CROT (CROT) were lysed prior to western blot analysis.

B) 30,000 cells stably expressing the indicated plasmid were plated into a 12-well plate. The next day, the wells were imaged (0hr time point), and then the media was changed for DMEM that lacks glucose. Cells were then imaged 12hr after starvation initiation and 24hr after starvation initiation. Representative images are shown.

C) Quantification of the experiment performed in **B**. At each time point, images were taken of five random fields of view. For each image, the number of living and dead cells were counted. The data presented represent the mean percent of living cells counted at each time point. Bars represent mean, points represent individual measurements, error bars represent standard deviation. Two-tailed unpaired t-tests were used to generate p-values. *p<0.05; **p<0.01.
